# Waldenstrom's Macroglobulinemia: A Report of Two Cases, One with Severe Retinopathy and One with Renal Failure

**DOI:** 10.1155/2017/3732902

**Published:** 2017-10-31

**Authors:** Naoko Kudo, Masakatsu Usui, Yukiharu Nakabo, Ken-ichi Yoshida, Kenji Miki, Takashi Osafune, Keisuke Nishimura, Shinsaku Imashuku

**Affiliations:** ^1^Division of Hematology, Takasago-seibu Hospital, Takasago 676-0812, Japan; ^2^Department of Internal Medicine, Uji-Tokushukai Medical Center, Uji 611-0042, Japan; ^3^The Center for Hematological Diseases, Takeda General Hospital, Kyoto 601-1495, Japan; ^4^Division of Ophthalmology, Takasago-seibu Hospital, Takasago 676-0812, Japan; ^5^Department of Urology, Uji-Tokushukai Medical Center, Uji 611-0042, Japan; ^6^Department of Pathology, Uji-Tokushukai Medical Center, Uji 611-0042, Japan; ^7^Department of Laboratory Medicine, Uji-Tokushukai Medical Center, Uji 611-0042, Japan

## Abstract

We report here two cases of Waldenstrom's macroglobulinemia (WM), one with central nervous system (CNS) symptoms and severe retinopathy and one with renal failure. In both cases, the serum IgM levels exceeded 3,000 mg/dL and monoclonal IgM-kappa was observed in the blood. At onset, Case 1, a 63-year-old female, developed CNS symptoms—namely, drowsiness and syncope. Case 2, a 58-year-old male, had nausea and dysgeusia on admission associated with renal failure, which is quite rare in patients with WM. Both patients exhibited hyperviscosity-related retinopathy, but it was particularly severe in Case 1: she suddenly lost her vision after admission. However, her vision recovered completely during treatment. Case 2 required hemodialysis immediately after admission. Needle biopsy of his kidney revealed tubulointerstitial nephritis with marked infiltration with CD20-positive lymphoplasmacytic lymphoma cells. After treatment, Case 1 has been in a remission longer than 8 years, but Case 2 died of pneumonia in 6 months. Since the initial symptoms of WM are ambiguous and vary significantly and hyperviscosity-related ophthalmological problems or severe renal dysfunction can arise, it is essential to promptly measure serum IgM levels and to institute appropriate care immediately when WM is confirmed in a patient.

## 1. Introduction

Waldenstrom's macroglobulinemia (WM) is a lymphoplasmacytic lymphoma (LPL) that is characterized by high levels of monoclonal immunoglobulin M (IgM) protein in the blood. WM is one of low-grade B-cell lymphomas [1, 2]. The incidence of LPL in Japan is very rare. In 1998, it was reported that 22 (0.7%) of all 3,194 malignant lymphomas diagnosed in Japan were LPLs. Since the vast majority of LPLs are WM, this indicates that WM is exceedingly uncommon in Japan [3]. A diagnosis of WM is made when the monoclonal IgM-kappa protein that associates with ≥10% clonal LPL cells in the bone marrow is detected [1]; however, this 10% cutoff seems not to be mandatory, because there are a number of patients with <10% bone marrow involvement with high-serum IgM and viscosity levels, necessitating therapy [1, 2]. Also, it is often difficult to diagnose WM morphologically because the lymphoplasmacytoid LPL cells in the bone marrow can resemble mature lymphocytes or plasma cells [4, 5]. In terms of immunophenotype, lymphoplasmacytoid LPL cells generally express CD19, CD20, and kappa light chain. In addition, CD38 and/or CD138 can be used to identify plasmacytoid lymphocytes [6]. Recent studies have shown that there are several genomic abnormalities that are characteristic for WM: the most common are the L265P mutation in MYD88 (found in 95–97% of patients with WM) and a somatic mutation in CXCR4 (found in 30–40% of patients with WM) [7]. These mutations (MYD88 and CXCR4) were mentioned to have the impact on treatment decisions on WM [8].

The symptoms of WM range from vague general symptoms such as fatigue, weight loss, and anorexia to more specific symptoms—namely, anemia/thrombocytopenia (due to LPL cell infiltration in the bone marrow), hyperviscosity (due to elevated serum levels of a high molecular weight IgM pentamer), and hyperviscosity-related neurological, vascular, and hemorrhagic symptoms. The neurological symptoms include dizziness, ataxia, paresthesia, and coma. The hemorrhagic symptoms include epistaxis, gingival and gastrointestinal hemorrhage, and menorrhagia: these symptoms arise from vascular disturbances [1, 2]. A particularly characteristic finding in WM is retinopathy (specifically, dilated retinal veins, retinal hemorrhage, and papilledema) [9, 10]. In addition, WM occasionally associates with nephropathy (it appears to be much more common in multiple myeloma). Nephropathy can arise via various mechanisms, including the infiltration of the kidneys with LPL cells [11]. Several prognostic risk factors have been identified for WM—namely, an *older* age, presence of anemia or thrombocytopenia, high levels of serum β_2_ microglobulin, and *high* monoclonal IgM concentrations. It is recommended to adjust the treatment for WM according to the presence of these risk factors [1, 2, 8]. In terms of eventual outcome, a number of highly effective therapeutic agents are now available for WM, and the prognosis is not as dire as it once was, but it still remains incurable [1, 12, 13]. We report here two cases of WM; one with CNS symptoms and severe retinopathy, and one with renal failure.

## 2. Case Report

### 2.1. Case 1

**Figure 1:**
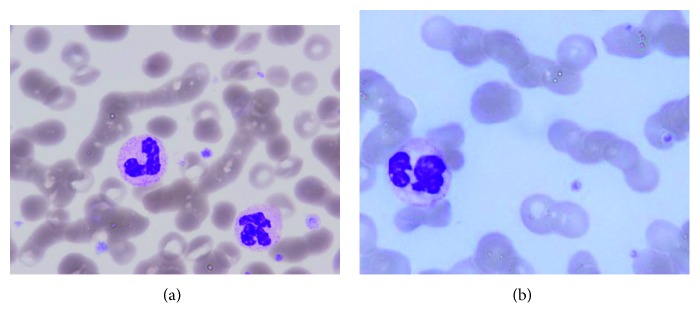
Peripheral blood smears from (a) Case 1 and (b) Case 2, which exhibit rouleaux formation of the red blood cells.

**Figure 2:**
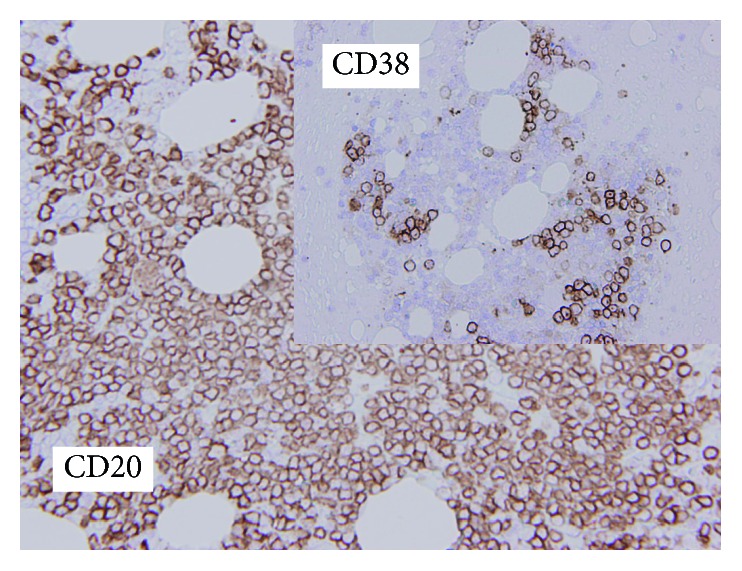
Immunostaining of lymphoplasmacytic lymphoma (LPL) cells in the bone marrow clot preparation from Case 1. The LPL cells were diffusely positive for CD20 but only sporadically positive for CD38 (original magnification, ×400).

**Figure 3:**
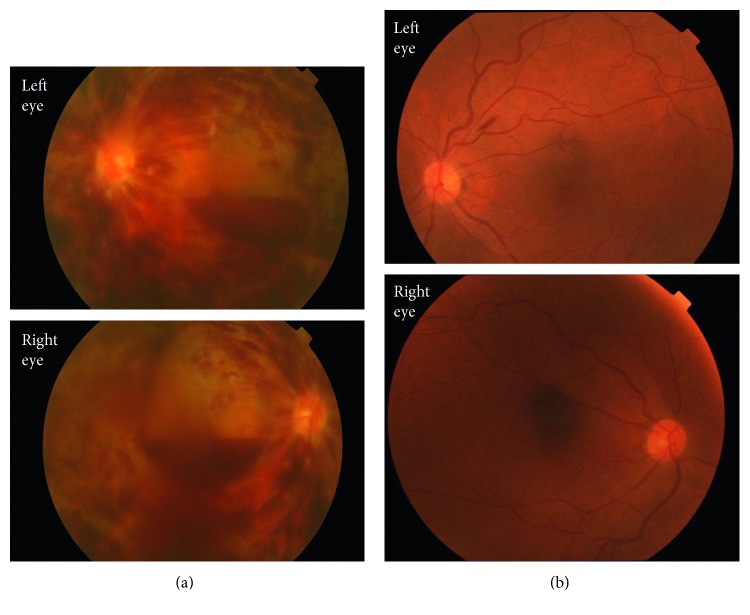
Fundoscopic findings. (a) Case 1 exhibited bilateral central retinal vein occlusion (CRVO) and optic nerve head edema with diffuse peripheral and central retinal hemorrhages that were accompanied by a large vitreous hemorrhage. (b) Case 2 exhibited bilateral tortuous blood vessels with a rod-like retinal hemorrhage (left eye) without macular edema.

A 63-year-old Japanese female was referred with complaints of lightheadedness and drowsiness. In the 2 weeks prior to admission, she had episodes of fainting at the shopping mall and in the bathroom at home. When she attended the clinic near her home, she was found to have high levels of serum IgM. On admission in our hospital, she was alert with no disorientation but exhibited slight consciousness impairment (Japan Coma Scale; I-1). Her BP was 130/86 mmHg, HR was 61 bpm, SpO_2_ was 95% (room air), and RR was 20/min. Her laboratory data are summarized in Table 1. A peripheral blood smear clearly exhibited rouleaux formation (Figure 1). Bone marrow was only available as a clot: smear preparations could not be obtained because of rapid coagulation at the time of bone marrow aspiration. As shown in Figure 2, the bone marrow was infiltrated with LPL cells that were diffusely positive for CD20 but only sporadically positive for CD38. The karyotypes of the bone marrow were not available. After admission and diagnosis of WM, the patient remained drowsy on and off. A week after admission, she developed a sudden loss of vision due to severe retinopathy (Figure 3) in association with nasal and genital hemorrhages. In the first 8 weeks after admission, Case 1 was given one course of double-filtration plasmapheresis, four courses of plasma exchange, and three doses of rituximab (375 mg/m^2^/dose, drip) associated with two courses of 2-chloro-2′-deoxyadenosine (2-CDA; 0.1 mg/kg/day × 5 days, drip) chemotherapy. This initial treatment was followed by monthly rituximab treatment. Fortunately, while on the initial treatment, the patient's vision recovered completely (4 weeks after the patient reported having loss of vision). Her serum IgM levels dropped gradually but constantly in response to the initial treatment and the following monthly rituximab treatment. After 6 months, her serum IgM levels were <500 mg/dL, and we started oral thalidomide (100 mg/day, daily) combined with rituximab every 4 months as the maintenance treatment. Eight years after the WM diagnosis, the patient has been in remission without any symptoms. She has normal serum IgM levels, a normal free light chain kappa/lambda ratio with residually detectable M protein in her blood.

### 2.2. Case 2

**Figure 4:**
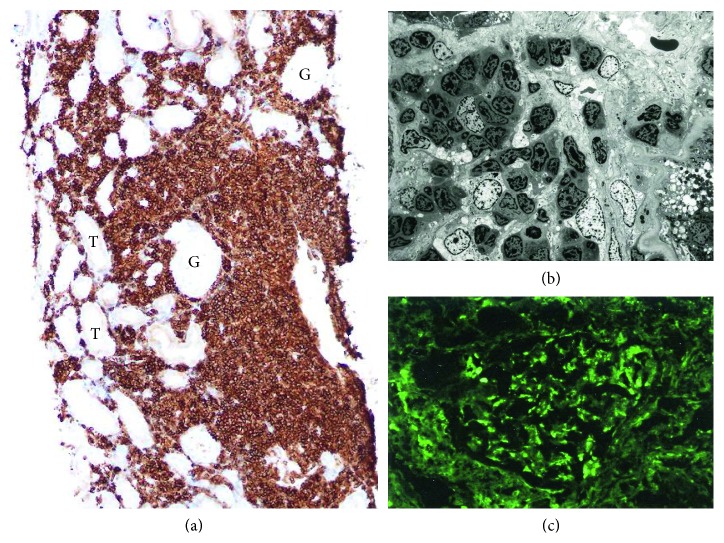
Histopathology findings of the biopsied tissue of the right kidney of Case 2. (a) Light microscopy revealed tubulointerstitial nephritis with marked infiltration by lymphoplasmacytic lymphoma (LPL) cells that were diffusely positive for CD20 (G: glomerulus; T: tubules; original magnification, ×100). (b) Electron microscopy showed that the renal tissue was infiltrated by LPL cells (original magnification, ×1,000). (c) Fluorescence microscopy revealed dot-like IgM depositions in the mesangial region (there were no depositions for IgG, IgA, or C3c). Significant morphological glomerular changes were not noted (photos not shown).

A 58-year-old Japanese male was transferred to the clinic near his home due to renal failure. Eight months prior to admission, his renal function was normal (serum BUN 14 mg/dL and creatinine 0.87 mg/dL) and he was not anemic (Hb 10.0 g/dL). One month prior to admission, the patient complained of nausea, loss of appetite, decreased water intake, dysgeusia, watery diarrhea, and oliguria. On admission, he was alert and afebrile, with a BP of 118/59 mmHg, a HR of 67 bpm, a SpO_2_ of 99% (room air), and a RR of 16/min. His laboratory data are summarized in Table 1. A peripheral blood smear clearly exhibited rouleaux formation (Figure 1). His bone marrow was infiltrated with LPL cells (32% of NCC) that were positive for CD19 (26.4%), CD20 (28.3%), and kappa light chain (38.3%). The bone marrow clot section contained aggregates of LPL cells that were positive for CD20 and CD138 (data not shown). The bone marrow karyotype was 46, XY (20/20). Other laboratory data, including the collagen disease markers for systemic lupus erythematosus, were all negative. Although the patient did not complain of vision problems, fundoscopy revealed bilateral tortuous blood vessels with a few hemorrhages but no macular edema (Figure 3). The patient's main problem was renal failure that was characterized by high serum levels of BUN (124.4 mg/dL), creatinine (11.1 mg/dL), and beta-2-microglobulin (beta-2-MG; 36.5 mg/dL). Given that the renal failure had led to hyperkalemia (7.6 mmol/L), oliguria, and metabolic acidosis (pH 7.110; BE: −17.9 mmol/dL), hemodialysis (three days a week) was introduced immediately. In addition, to clarify the pathogenesis of the renal failure, a needle biopsy of the right kidney was performed on day 12 of admission. Light and electron microscopic studies of the biopsied kidney tissue revealed massive interstitial LPL cell infiltration, while the fluorescence microscopic study revealed mild dot-like mesangial IgM depositions (Figure 4). Given his relatively young age and presumed poor outcome, the patient was sent to a tertiary referral center for renal failure care and chemotherapy. At the referral center, BR chemotherapy, a combination of rituximab (375 mg/m^2^, drip, day 1) and bendamustine (90 mg/m^2^, drip, days 2 and 3) q 4 weeks, was started with continuation of hemodialysis. After 3 courses of a total of 6 courses of BR chemotherapy planned, the patient's condition was stable with serum creatinine 4.57 mg/dL and IgM 1,246 mg/dL, showing gradual improvement in renal function; however, considering the reduction rate of serum creatinine, it seemed not to be possible for the patient to make a successful hemodialysis withdrawal. Unfortunately, the patient died of severe pneumonia during maintenance dialysis after the sixth cycle of rituximab/bendamustine chemotherapy.

## 3. Discussion

The clinical features of WM vary from anemia/thrombocytopenia that is due to LPL infiltration in the bone marrow to CNS symptoms that are due to the hyperviscosity, which is caused by the high serum IgM concentrations. In particular, the thickened blood causes poor brain circulation, which leads to CNS problems such as confusion, dizziness, and syncope: these CNS symptoms were all apparent in our Case 1. Another sign of the hyperviscosity that characterizes WM is vision problem such as blurred vision or blindness: this reflects the fact that hyperviscosity impairs the circulation in the retina and causes hemorrhages around the small retinal vessels [9, 10, 14]. In addition, although renal problems are uncommon in WM (∼5%) (unlike in multiple myeloma, where it occurs in 30–40% of patients), nephropathy (namely, nephrotic syndrome, renal failure, and uremic encephalopathy) can arise in patients with WM [11, 15]. Below, we will discuss three issues—namely, WM-related retinopathy, WM-related nephropathy, and the recent progress in the management of WM.

WM-related eye diseases vary from bilateral simultaneous central retinal vein occlusion (CRVO) [9, 10] and bilateral serous macular detachment [16] to LPL infiltration of the optic nerve [17]. However, vision loss in WM is mostly caused by bilateral simultaneous CRVO that is caused by hyperviscosity: it is estimated to affect 15–17% of all patients with WM [9, 18]. Notably, when Menke et al. studied the funduscopic findings in 46 patients with WM, they were able to classify them into three groups depending on the degree of the retinopathy. Thus, group 1 had no signs of retinopathy (52%), group 2 exhibited dilated retinal veins with peripheral retinal hemorrhages (39%), and group 3 had optic disc edema, peripheral and central retinal hemorrhages, and venous sausaging (9%). Menke et al. found that the mean serum IgM level associated with the first retinal changes in WM was 5,442 mg/dL (range: 2,950–8,440 mg/dL) [14]. Indeed, our Case 1, who developed blindness due to the group 3–type renal damage defined by Menke et al., had IgM levels of 6,080 mg/dL at the time the blindness arose. Moreover, our Case 2 showed mild retinopathy when his serum IgM level was 3,603 mg/dL. These observations suggest that when patients exhibit bilateral simultaneous CRVO and hyperviscosity, their serum IgM levels must be determined. In addition, when a patient with WM is diagnosed, they should undergo routine funduscopic evaluation. It is possible to reverse retinopathy and reduce the abnormal venous dilatation in WM by plasmapheresis [19]. However, it has been noted that even when multiple plasmapheresis treatments are given, serous macular detachments can persist [16]. Nevertheless, in our Case 1, her severe retinopathy was successfully cured by her treatment, which consisted of four courses of plasma exchange, rituximab, and 2-CDA chemotherapy.

Various monoclonal IgM-secreting lymphoproliferative diseases, including WM, are associated with renal lesions [11, 20, 21]. According to Vos et al., the cumulative incidence of renal involvement in WM is 5.1% in 15 years [11]. Recently, Chauvet et al. [21] examined 35 patients with renal damage that related to B-cell lymphoproliferative disease involving monoclonal IgM secretion. Of the 35 patients, 26 had WM. Chauvet et al. were able to divide the 35 patients into three groups on the basis of their renal pathology. Thus, group 1 had glomerular AL amyloidosis (31.4%), group 2 had nonamyloid glomerulopathies (42.8%), and group 3 had tubulointerstitial nephropathies (25.7%: this included three cases of LPL infiltration). The study of Vos et al. on the WM-related nephropathy also showed three major similar groups on the basis of their renal pathology; 25% had group 1–type renal damage (amyloidosis), 23% had group 2–type renal damage (monoclonal IgM deposition disease/cryoglobulinemia), and 18% had group 3–type renal damage (tubulointerstitial LPL infiltration), and the remaining consisted of other types of renal damage [11]. In addition, in an analysis of eight cases of WM nephropathy by Audard et al. [20], three patients had tubulointerstitial LPL infiltration (i.e., group 3–type renal damage; 37.5%). In terms of symptoms, Chauvet et al. [21] reported that nephrotic syndrome was mostly observed in groups 1 and 2, whereas acute kidney injury and/or proximal tubular dysfunction were noted in group 3. In our Case 1, nephropathy did not arise despite the fact that she had proteinuria on admission. In our Case 2, it was difficult to determine whether he had acute renal failure or not. However, the kidney damage did progress quite rapidly: he had normal kidney function only 8 months prior to his admission for renal failure. The main cause of his renal failure was tubulointerstitial LPL infiltration (i.e., he had group 3–type renal damage; Figure 4). However, the impact of the mild mesangial IgM deposition that was observed is unknown. In terms of response to therapy by patients with biopsy-confirmed monoclonal IgM nephropathy, Chauvet et al. [21] reported that five of ten evaluable group 1 patients, nine of fifteen evaluable group 2 patients, and five of eight evaluable group 3 patients exhibited a renal response to first-line treatment. In addition, Vos et al. [11] showed that the median overall survival of WM patients with nephropathy was 11.5 years, which was shorter than the overall survival of the WM patients without nephropathy (16 years, *P*=0.03). However, the patients with nephropathy whose renal function remained stable or improved after treatment had better overall survival than those whose nephropathy worsened despite treatment (*P*=0.05). Unfortunately, in our Case 2, the renal damage was progressed prior to the initiation of chemotherapy; thus, it seemed not to be possible for the patient to make a successful hemodialysis withdrawal.

Regarding the management of WM, it was proposed that initiation of therapy can be based on symptoms such as recurrent fever, night sweats, fatigue, anemia, or weight loss rather than on the serum IgM levels. In addition, symptoms due to hyperviscosity syndrome, renal insufficiency, or symptomatic cryoglobulinemia may also be indications for therapy initiation [22]. Besides effective plasmapheresis in the management of hyperviscosity [19], chemotherapy with a combination of rituximab and 2-chloro-2′-deoxyadenosine (2-CDA), or of rituximab and bendamustine, has been proven effective for WM [23–25]. This was confirmed by the success of rituximab/2-CDA combination therapy in our Case 1 as well as a partial response of rituximab/bendamustine therapy in our Case 2. We also successfully managed Case 1 during the maintenance therapy with a combination of rituximab and thalidomide. More recent treatment recommendations in the international consensus paper for WM include other novel and promising agents such as ofatumumab, lenalidomide, bortezomib, and the BTK inhibitor ibrutinib. [8]. However, because of his early death, our Case 2 lost the chance of receiving such novel therapeutic measures.

In summary, we presented two cases of WM; one with CNS symptoms and severe retinopathy, and one with renal failure that was due to tubulointerstitial infiltration of LPL cells (as confirmed by renal biopsy). To obtain excellent outcomes in patients with WM, it is essential that ophthalmological and/or renal problems are early diagnosed and appropriately managed.

## Figures and Tables

**Table 1:** Clinical features and laboratory data.

	Case 1	Case 2
Age (yrs)/gender	63/F	58/M
Chief complaints	Lightheadedness	Nausea
	Drowsiness	Dysgeusia
	Blurred vision	Oliguria
Major clinical features	Retinopathy (group 3^∗^)	Renal failure (group 3^∗∗^)
*Laboratory data on admission*		
WBC (/*µ*L)	6,900	8,200
Hb (g/dL)	7.9	8.2
Platelet counts (/*µ*L)	124,000	107,000
PB smear	Rouleaux formation	Rouleaux formation
CRP (mg/dL)	0.19	1.15
LDH (U/L)	174	102
Total protein (g/dL)	9.2	9.3
Albumin (g/dL)	2.1	3.2
Serum BUN (mg/dL)	20.9	124.4
Creatinine (mg/dL)	1.25	11.1
Na (mmol/L)	135	137
K (mmol/L)	4.3	7.6
Cl (mmol/L)	98	112
IgG (mg/dL)	427	473
IgA (mg/dL)	137	20
IgM (mg/dL)	6,080	3,603
Beta-2-MG (mg/dL)	4.0	36.5
sIL-2R (U/L)	767	NT
Monoclonal protein	IgM-kappa	IgM-kappa
Proteinuria	2+	2+
MYD88	NT	NT
*Treatment*		
Initial management and chemotherapy induction	DFPP/PE/rituximab/2-CDA	Hemodialysis
		Rituximab/bendamustine
Maintenance	Rituximab/thalidomide	None
*Outcome*		
Alive (yrs)	8+	0.5^∗∗∗^

^∗^Defined by Menke et al. [14]; ^∗∗^defined by Chauvet et al. [21], Vos et al. [11], and Audard et al. [20]; ^∗∗∗^died of severe pneumonia after the sixth cycle of rituximab/bendamustine. NT = not tested, MYD88 = myeloid differentiation 88, DFPP = double-filtration plasmapheresis, PE = plasma exchange, 2-CDA = 2-chloro-2′-deoxyadenosine.

## References

[1] Gertz M. A. (2017). Waldenström macroglobulinemia: 2017 update on diagnosis, risk stratification, and management. *American Journal of Hematology*.

[2] Castillo J. J., Garcia-Sanz R., Hatjiharissi E. (2016). Recommendations for the diagnosis and initial evaluation of patients with Waldenström macroglobulinaemia: a task force from the 8th International Workshop on Waldenström macroglobulinaemia. *British Journal of Haematology*.

[3] Lymphoma Study Group of Japanese Pathologists (2000). The World Health Organization classification of malignant lymphomas in Japan: incidence of recently recognized entities. *Pathology International*.

[4] Morice W. G., Chen D., Kurtin P. J., Hanson C. A., McPhail E. D. (2009). Novel immunophenotypic features of marrow lymphoplasmacytic lymphoma and correlation with Waldenström’s macroglobulinemia. *Modern Pathology*.

[5] Berger F., Traverse-Glehen A., Felman P. (2005). Clinicopathologic features of Waldenstrom’s macroglobulinemia and marginal zone lymphoma: are they distinct or the same entity?. *Clinical Lymphoma*.

[6] Kyrtsonis M. C., Levidou G., Korkolopoulou P. (2011). CD138 expression helps distinguishing Waldenström’s macroglobulinemia (WM) from splenic marginal zone lymphoma (SMZL). *Clinical Lymphoma Myeloma Leukemia*.

[7] Hunter Z. R., Yang G., Xu L., Liu X., Castillo J. J., Treon S. P. (2017). Genomics, signaling, and treatment of Waldenström macroglobulinemia. *Journal of Clinical Oncology*.

[8] Leblond V., Kastritis E., Advani R. (2016). Treatment recommendations from the Eighth International Workshop on Waldenström’s macroglobulinemia. *Blood*.

[9] Alexander P., Flanagan D., Rege K., Foss A., Hingorani M. (2008). Bilateral simultaneous central retinal vein occlusion secondary to hyperviscosity in Waldenstrom’s macroglobulinaemia. *Eye*.

[10] Chanana B., Gupta N., Azad R. V. (2009). Case report: bilateral simultaneous central retinal vein occlusion in Waldenström’s macroglobulinemia. *Optometry*.

[11] Vos J. M., Gustine J., Rennke H. G. (2016). Renal disease related to Waldenström macroglobulinaemia: incidence, pathology and clinical outcomes. *British Journal of Haematology*.

[12] Castillo J. J., Olszewski A. J., Kanan S., Meid K., Hunter Z. R., Treon S. P. (2015). Overall survival and competing risks of death in patients with Waldenström macroglobulinaemia: an analysis of the Surveillance, Epidemiology and End Results database. *British Journal of Haematology*.

[13] Abeykoon J. P., Yanamandra U., Kapoor P. (2017). New developments in the management of Waldenström macroglobulinemia. *Cancer Management Research*.

[14] Menke M. N., Feke G. T., McMeel J. W., Branagan A., Hunter Z., Treon S. P. (2006). Hyperviscosity-related retinopathy in Waldenstrom macroglobulinemia. *Archives of Ophthalmology*.

[15] Dimopoulos M. A., Terpos E., Chanan-Khan A. (2010). Renal impairment in patients with multiple myeloma: a consensus statement on behalf of the International Myeloma Working Group. *Journal of Clinical Oncology*.

[16] Pilon A. F., Rhee P. S., Messner L. V. (2005). Bilateral, persistent serous macular detachments with Waldenström’s macroglobulinemia. *Optometry and Vision Science*.

[17] Nishida H., Hashida R., Hatano M., Hori M., Obara K. (2014). Optic nerve involvement of Waldenström’s macroglobulinemia: with autopsy findings. *Neurological Sciences*.

[18] Merlini G., Baldini L., Broglia C. (2003). Prognostic factors in symptomatic Waldenström’s macroglobulinemia. *Seminars in Oncology*.

[19] Menke M. N., Feke G. T., McMeel J. W., Treon S. P. (2008). Effect of plasmapheresis on hyperviscosity-related retinopathy and retinal hemodynamics in patients with Waldenstrom’s macroglobulinemia. *Investigative Ophthalmology & Visual Science*.

[20] Audard V., Georges B., Vanhille P. (2008). Renal lesions associated with IgM-secreting monoclonal proliferations: revisiting the disease spectrum. *Clinical Journal of American Society of Nephrology*.

[21] Chauvet S., Bridoux F., Ecotière L. (2015). Kidney diseases associated with monoclonal immunoglobulin M-secreting B-cell lymphoproliferative disorders: a case series of 35 patients. *American Journal of Kidney Diseases*.

[22] Kyle R. A., Treon S. P., Alexanian R. (2003). Prognostic markers and criteria to initiate therapy in Waldenstrom’s macroglobulinemia: consensus panel recommendations from the Second International Workshop on Waldenstrom’s Macroglobulinemia. *Seminars in Oncology*.

[23] Dimopoulos M. A., Kastritis E., Roussou M. (2009). Rituximab-based treatments in Waldenström’s macroglobulinemia. *Clinical Lymphoma and Myeloma*.

[24] Laszlo D., Andreola G., Rigacci L. (2010). Rituximab and subcutaneous 2-chloro-2′-deoxyadenosine combination treatment for patients with Waldenstrom macroglobulinemia: clinical and biologic results of a phase II multicenter study. *Journal of Clinical Oncology*.

[25] Varettoni M., Marchioni E., Bonfichi M. (2015). Successful treatment with rituximab and bendamustine in a patient with newly diagnosed Waldenström’s macroglobulinemia complicated by Bing-Neel syndrome. *American Journal of Hematology*.

